# Senescence program and its reprogramming in pancreatic premalignancy

**DOI:** 10.1038/s41419-023-06040-3

**Published:** 2023-08-17

**Authors:** Kailing Yang, Xiaojia Li, Keping Xie

**Affiliations:** 1 Center for Pancreatic Cancer Research, The South China University of Technology School of Medicine, Guangzhou, China; 2 The South China University of Technology Comprehensive Cancer Center, Guangdong, China; 3 The Second Affiliated Hospital and Guangzhou First People’s Hospital, South China University of Technology School of Medicine, Guangdong, China

**Keywords:** Pancreatic cancer, Oncogenesis

## Abstract

Tumor is a representative of cell immortalization, while senescence irreversibly arrests cell proliferation. Although tumorigenesis and senescence seem contrary to each other, they have similar mechanisms in many aspects. Pancreatic ductal adenocarcinoma (PDA) is highly lethal disease, which occurs and progresses through a multi-step process. Senescence is prevalent in pancreatic premalignancy, as manifested by decreased cell proliferation and increased clearance of pre-malignant cells by immune system. However, the senescent microenvironment cooperates with multiple factors and significantly contributes to tumorigenesis. Evidently, PDA progression requires to evade the effects of cellular senescence. This review will focus on dual roles that senescence plays in PDA development and progression, the signaling effectors that critically regulate senescence in PDA, the identification and reactivation of molecular targets that control senescence program for the treatment of PDA.

## Introduction

PDA is one of the deadliest malignancies with lowest 5-year survival rate. PDA patients are usually at clinically terminal stages, resistance to chemotherapy and radiotherapy [1]. Understanding the mechanisms that leading to pancreatic preneoplasms is essential to improve early diagnosis and treatment strategies [2]. The pathogenesis of PDA involves a variety of factors, including inflammation and gene mutation [3]. In response to environmental stress, various cell types change their identity, transform into ductal-like cells [4]. Acinar cells are the largest component of pancreas, and a predominsant cell source for PDA [5].

Senescence is closely involved in PDA development and progression. The first formal description of senescence is back to the 1960s, when Hayflflick and colleagues observed that normal human diploid fibroblasts had limited proliferative capacity in culture [6]. It is now clear that this permanent growth arrest, termed cell senescence, is a program executed or activated by cellular response to many kinds of stress, including attrition of telomeres, DNA damaging agents, oxidative stress, oncogene activation, and others [7]. What happens to senescent cells in the process of cancer development remains unclear. Evidently, senescent cells are abundant in precancerous lesions, but rare in cancer [8, 9]. Premalignant cells can bypass senescent responses, eventually develop into malignant aggressive tumors [10]. Importantly, senescent cells release a series of cytokines, collectively called senescence-associated secreted phenotype (SASP), which can either be cleared by immune cells or create a microenvironment stimulating tumor growth and promoting tumor invasion and metastasis [11, 12].

## Hallmarks and mechanisms of senescence

**Fig. 1:**
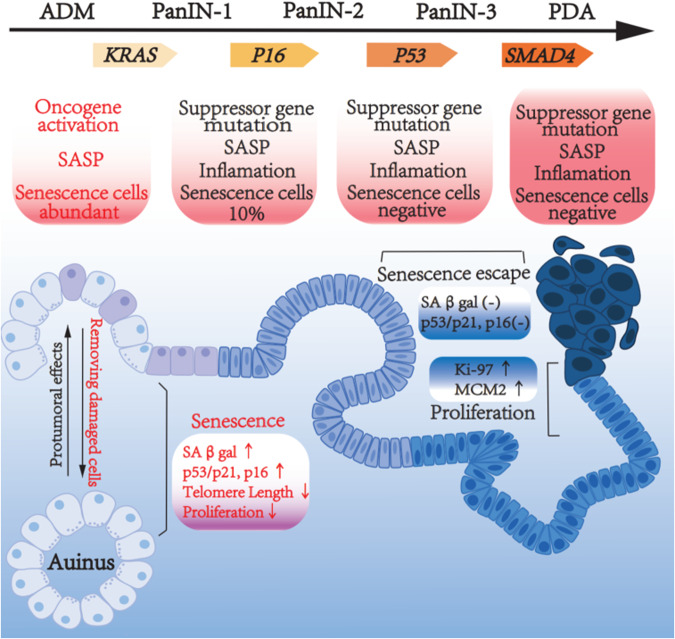
Senescence in PDA initiation. Hallmarks of senescent cells include growth arrest, expression of p53, p21, p16, SAβgal, and robust secretion of SASP. Senescence plays a vital role in the process of ADM-PanIN-PDA progression. Majority of ADM undergo senescence, eliminating pre-malignant cell. Some ADM and PanIN contribute to oncogenesis, while successful progression of ADM to PDA requires escape senescence.

Cellular senescence is accompanied by striking morphological, biochemical, and functional transformation, e.g., flattened, enlarged, and multinucleated morphology and mitochondrial reduction [7, 11, 12]. Senescence response ensures cell cycle arrest, rendering cell incapable of forming a tumor [13]. Senescent cells are generally identified by several features and molecular markers. Some of these markers reflect the activation of mechanisms that attribute to the senescence program [14]. The best markers of cellular senescence should provide insight into the fundamental causes of senescence (Fig. 1).

### Induction of senescence-associated β-galactosidase activity

Senescence-associated β-galactosidase (SAβgal) is the most extensively utilized senescence biomarker [15]. The enzyme activity of β-galactosidase displays high at pH 4 in normal cells, while SAβgal activity can be detectable at pH 6.0 in senescence cells [16]. Enhanced activity of SAβgal occurs as senescence continues [17]. Despite the SAβgal is the most reliable and specific marker for senescence, the increase of SAβgal is an outcome of senescence rather than a cause [15, 18]. SAβgal induction partly originates from lysosomal β-galactosidase gene (*GLB1*) [18].

### Telomere shortening

Telomere shortening is the main trigger of senescence. As a marker, change of telomere is consistent and overlapping with a majority of other senescence-associated cellular markers [19]. The cell cycle is divided into four stages in eukaryotes, the gap before DNA replication (GI), the DNA synthetic phase (S), the gap after DNA replication (G2), and the mitotic phase (M) [20]. Normal cell division is finite and cells can permanently withdraw from the cell cycle, due to the unidirectional nature of DNA polymerases and failure to replicate the lagging strands, telomeric DNA loses with each S phase. Cells with extremely shortened telomeres become senescent, and telomere erosion triggers the DNA damage response (DDR), which keeps the cell in a growth arrest state [19]. Consequently, telomere length is a marker of replicative capacity and senescence [21]. Cancer cells maintain telomeres for overcoming senescence and acquiring immortality [22]. Critically shortened telomeres and associated chromosomal instability contribute to neoplastic transformation, which allows continued tumor growth [23].

### p53/p21

p53 functions as a tetrameric transcription factor play a key role in the control of cell-cycle as well as DNA repair, apoptosis, and cellular stress responses. p53 either temporarily inhibits cell proliferation, activates DNA repair mechanisms, and induces cell death when damaged beyond repair, or pushing cells toward replicative senescence, a permanent arrest of proliferation [24]. Excessive activity of p53 can generate premature aging in variety of tissue types in mice [25]. The inhibition of p53 results in the re-entry of senescent fibroblasts into the cell cycle and immortalization, suggesting that onset and maintenance of cell senescence depend on p53 [26, 27]. *P53* is an important tumor suppressor gene, which can be activated by abnormal proliferation and DNA damage [27]. When the cells proliferate abnormally, the p19^ARF^ (called p14^ARF^ in humans) protein in the upstream of p53 directly binds to the MDM2 protein, a primary negative endogenous regulator of p53, and inhibits the degradation of p53 [28]. Its continuous activation makes the cell enter the irreversible growth arrest state of cell senescence.

Another marker commonly used to identify senescent cells is p21 [17]. p21 is an important target downstream of p53 [29]. p21 can effectively block cell cycle progression by keeping pRb from becoming phosphorylated and inactivated [30]. Consequently, p21 inactivates proliferating cell nuclear antigen during early stage of senescence [31]. It is transient that p53 and p21 are activated in senescent cells. p53 and p21 protein levels decline after growth arrest is established. When p21 expression decreases, p16^Ink4A^ (or p16 hereafter), another CDK inhibitor, constitutively increases, thus maintaining growth arrest [32].

### p16^INK4a^

As one member of the INK4 family, INK4a encodes two distinct proteins, p16^INK4a^ and p19^ARF^, molecular twins that have their own promoter sequences, and each can inhibit cell growth at different points in the cycle and via apparently distinct mechanisms [33, 34]. p19^ARF^ achieves cell-cycle arrest by stabilizing the p53 protein, and mice lacking p19^ARF^ develop tumors early in life [35]. p16 is an inhibitor of cyclin D-dependent CDK4 and CDK6 complexes. An important feature of senescence is attributable to highly dynamic expression of p16: not only normal expression in adult tissues [35–37], but also high expression in senescent cells under different stress situations [38]. The p16/Rb is a key signaling pathway and essential for regulating senescence program. Expression of p16 inhibits CDK4/6-mediated phosphorylation of Rb, generates G1 cell cycle arrest and ultimately causes cells to enter a senescent state [39]. The increased p16 is a part of multistep process that is turned on in senescence. p16 accumulates with SAβgal activity and cell volume [31], and significantly upregulates with advanced age [25]. Indeed, *P16* functions as a tumor suppressor through its role as a principal mediator of cellular senescence, and approximately half of cancers exhibit its inactivation, which is originally identified as tumor suppressor gene [39–41].

### SASP

Although proliferative pathways are terminally shutdown, senescent cells remain metabolically active and produce an intricate secretome known as SASP [42]. The components of SASP are variable and up to the triggers of senescence [43]. Various components of the SASP can transmit senescence signals to the neighboring non-senescent cells and induce paracrine senescence [44]. Therefore, senescence can affect the microenvironment through paracrine mechanisms. SASP usually has double effects. On the one hand, SASP can reinforce senescence, promote the repair of damaged tissues [45], and recruit the immune system against tumor development and progression [46]. On the other hand, SASP is also involved in tumorigenesis by creating an inflammatory microenvironment, destroying the normal structure of the tissue, and inducing the epithelium-mesenchymal transitions (EMT), thus promoting tumor growth and metastasis [47].

## Activation of senescence in pancreatic premalignancy

Senescence exists in precancerous lesions but not in similar lesions that have already developed into aggressive cancer. Human melanocytic nevi undergo BRAFV600E-induced senescence, maintain the cell growth stagnant, and rarely deteriorate into melanoma cells [9]. In mouse models of lung tumors, oncogene-induced markers of senescence are expressed in lung adenomas, while few lung adenomas progress into adenocarcinomas [48]. Likewise, prostate tumors [49], colon adenomas [50], astrocytomas [51], and neurofibromas [52] also express senescence markers in their early stages. Therefore, senescence has historically been viewed as a mechanism of tumor suppression, inhibiting uncontrolled cell proliferation. Senescence has a significant role in the pathogenesis of PDA, particularly in early stage. At the non-invasive precursor stage of PDA development, senescence program is a critical obstacle, limiting PDA initiation and progression [48, 53].

### Senescence in ADM

**Fig. 2:**
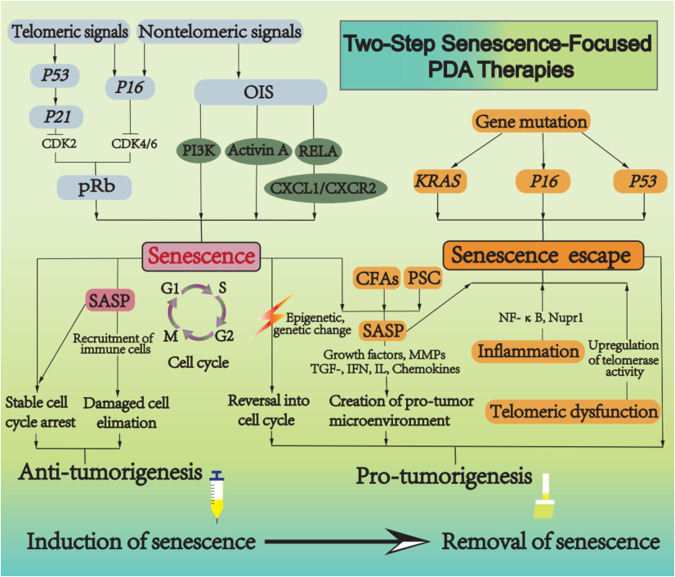
The key pathways and double-edged sword effects of senescence. Telomeric signals and nontelomeric signals can trigger senescence. Induction of p53 and p16 by senescence-inducing signals restrains CDK2 and CDK4/6, causing phosphorylation of pRb and G1 cell cycle arrest. Senescence can prevent premalignant progression. During PDA development, the senescent microenvironment cooperates with multiple factors and significantly contributes to tumorigenesis. Moreover, a series of genetic changes, e.g., *KRAS*, *P16,* and *P53* mutations, telomeric dysfunction, and pancreatitis, block the senescence, accelerating PDA progression. Pro-senescence followed by senescent cell removal could be a promising strategical option to treat PDA.

Acinar to ductal metaplasia (ADM), which arises under inflammatory conditions, is a pancreatic preneoplastic lesion that precedes the formation of pancreatic intraepithelial neoplasias (PanIN) [54]. ADM shows higher contents of SAβgal, p21, and p53 than PanIN [55]. Mice with pancreatitis induced by PDL or caerulein exhibit different degrees of activation of senescence program [56]. Genetically engineered mouse models (GEMM) develop PDA in animals by introducing specific genetic mutations in pancreas and mimic human PDA [57]. The original GEMM, known as KC mice, presented with the endogenous expression of oncogenic *LSL-Kras*^*G12D*^ in progenitor cells of the mouse pancreas. This model mimicks PDA progression from preinvasive neoplasias to invasive and metastatic disease [58]. In KC model, most ducts were normal at age of 12 weeks. As the animals age, the total number of ADM and PanIN clearly increased, high-grade lesions were observed at age of 5 months. It takes at least 6.25 months to go from PanIN to aggressive and invasive PDA [59]. Strikingly, in KC mice, both ADM and PanIN were positive for SAβgal and expressed abundant p21 and p53 proteins [55]. Evidently, senescence acts as a protection, hindering Kras activation and the cells with sporadic *Kras* mutation are eliminated through cell senescence in the early stage of PDA [60]. It is possible that the decline in proliferation rate of acinar and islet cells as compared to PanIN is owing to Ras-induced senescence [59, 61]. Senescent cells are observed frequently in PanIN-1 from pancreas-specific Kras^G12D^ mice, but rarely in PanIN-2/3 and PDA [62]. Kras activates senescence program via various signals. (1) PI3K, downstream of Kras, activation of PI3K signaling in the pancreas is sufficient to induce the formation of ADM and PanIN. ADM and PanIN are positive for SAβgal. These lesions also exhibit the activation of p16 and p53/p21 pathways. (2) In GEMM, Kras mediates senescence by activating the expression of activin A in ADM [63]. Activin A is the primary ALK4 ligand that drives PDA initiation, acts as a beneficial senescence-secreted factor produced by OIS during ADM. Activin A does not directly alter the formation of ADM, but inhibits the proliferation of ADM and limits the expansion and proliferation of PDA cells through the modulation of p16 or p21 expression, which critically impacts senescence among preneoplastic ADM cells [63]. (3) CXCL1 and CXCR2 ligand are necessary to induce senescence. After *Kras* mutation, the subunit RELA of NF-kB promotes senescence via the CXCL1/CXCR2 axis, thus inhibiting the progression of PDA precursor lesions [64]. Signal transduction cascades activated by Kras, p16, and p53 facilitate the transition from G0 or G1 phase to S phase in the cell cycle [65] (Fig. 2).

### Senescence in PanIN

In the pancreas, induction of senescence underlies the resistance of exocrine cells to robust oncogenic insults-mediated transformation, thereby preventing tumor development and progression [66]. PDA progresses from precursor PanIN lesions, which progress in a stepwise manner from grade 1 to grade 3 [67, 68]. Telomere shortening is nearly universal in PanIN [69, 70]. A low level of proliferation is an important feature of senescence. Compared with normal ducts, PanIN lesions display high levels of p53, p21, and Igfbpb7, another senescence marker. The expression of two markers of proliferation, Ki67 and the replication-licensing protein MCM2, is rarely observed in PanIN lesions, whereas their levels of expression increase in PDA [66, 71]. The number of senescent cells gradually decreases during the low-grade PanIN progression into high-grade PanIN [55, 66]. Based on the staining positivity of senescence-associated SAβgal and p16, 10% of PanIN-1 cells are senescent, when *Kras* mutations already exist, but prior to the senescence-associated tumor suppressor genes mutations [55]. High-grade PanIN-2/3 lesions are negative for senescence markers, including the expression of SAβgal and p16 [55, 66].

Not all KC mice can develop PDA as *Kras* mutation alone is not sufficient to induce PDA [59]. To promote the malignancy of pretumor lesions, a more robust transgenic mouse model was generated by concomitant *Kras*^*G12D*^ and *Trp53* mutations (known as KPC mice), which reconstructed the whole spectrum of PDA progression. By the age of 8–10 weeks, KPC mice harbored precursor lesions, and developed locally invasive tumors around three to six months [72]. Compared to the KC mice, the KPC mice develop advanced PDA with complete penetration in a shorter period of time, and recapitulate many of the salient clinical features that closely resemble the human disease. In vivo imaging and senescence marker staining demonstrated that majority of Kras-expressing pancreatic cells were not retained in the tissue, and those that were retained formed senescent PanIN lesions that rarely progressed to carcinomas. Loss of p53 allows the retention of Kras^G12D^-expressing cells, overcomes senescence and promotes metastasis, contributing to the rapid development of these premalignant lesions into PDA [71]. Initial events before developing into invasive cancer constitute a long window, when senescence is active in premalignant and diminishes once progression to invasive cancer. Induction of G0/G1 arrest is sufficient to convert PDA cells to a quiescent state, and greatly diminishes the malignancy potential of PDA cells [73]. Therefore, senescence plays a key role in preventing malignant progression [74].

### Oncogene-induced senescence

The presence of tumorigenesis barriers can slow or inhibit the precursor lesions from developing into mature malignant cancers. One of these barriers is DNA replication stress, which results in permanent cell proliferation arrest or apoptosis arrest [53]. The second barrier is mediated by oncogene-induced senescence (OIS). OIS is that the induction of senescence by oncogene activation leads to the withdrawal of cells from the cell cycle and prevents their uncontrolled proliferation [75]. Many studies have reported that OIS is a tumor suppressor mechanism in vivo. Abundant cells in premalignant tumors undergo OIS. Cancer cells do not enter this state of growth stagnation and proliferate indefinitely. As a major oncogene that causes PDA, *KRAS* mutations can cause abnormal cell proliferation. However, *KRAS* generally fails to convert precursor lesions into invasive cancers owing to triggering cellular senescence [76]. If mutated *KARS* is expressed in cells with intact cell cycle checkpoints, it can trigger OIS resulting in cell cycle arrest in the G1 phase, thus preventing further tissue transformation [77]. Notably, OIS appears to be further enhanced with increased simultaneous expression of p16 and p53, whereas *Kras* cooperates with genetic inactivation of those key senescence signaling pathways to accelerate tumorigenesis [66], highlighting the protective effect of senescence against tumor progression [78].

### Collateral effects of senescence

Despite the blockade of cell proliferation, senescent cells remain metabolically active and influence their environment. NF-κB and p38 MAPK pathways are activated in response to DNA damage and secrete SASP, including immunostimulatory cytokines and chemokines that recruit lymphocytes and metalloproteinases (MMP-7, 9 and 12) and regulate tumor formation [53, 79, 80]. Senescence can be transmitted to surrounding cells through SASP, which enhances the senescence program and recruits the immune cells [44, 81]. Primary and paracrine secondary senescence contributes to immune surveillance and elimination of damaged cells. Intercellular communication in senescent cells crucially influences surrounding cells, while secreted factors play predominant roles. Senescent cells can also affect neighboring cells through direct intercellular protein transfer (IPT) to regulate immune surveillance and impact tumorigenesis. Protein transfer to NK and T cells is increased in mouse preneoplastic pancreas, where senescent cells are present [82].

## Senescence reprogramming and PDA progression

Despite the protective role of senescence in premalignant lesions, accumulating evidence has underpinned that senescence correlates with accelerated PDA progression [83]. While senescence is involved in tumor suppression, it is unclear how PDA progression ensues in premalignant lesion. Presumably, it could occur through the following scenarios: (1) inactivation of the senescence allows preneoplastic lesions progress to neoplasia [84]; (2) senescence creates SASP, which along with the immune cells generates an inflammatory microenvironment for tumor growth [83]; and (3) senescent cells are reversed into the cell cycle and regain proliferation capacity.

### Senescence escape

Metaplasia of pancreatic acinar cells is plastic, and acinar cells are more sensitive to adverse stimuli than other cell types in the pancreas, which can resist transformation by genetic and/or environmental pressure. ADM is a reversible process if the cell stress can be resolved quickly [85]. However, acinar cells exposed to sustained stress or injury, particularly in the limited bouts of non-acute pancreatitis or presence of mutant *Kras*, may progress to PanIN and PDA [66]. Moreover, successful progression of ADM towards PDA requires bypassing senescence, since ADM and PanIN lesions display markers of cellular senescence [86]. PanIN lesions contain senescent, proliferating, and apoptotic cells, within positive staining of a subset of cells for SAβGal, p16, and p53, while 30% of cells stained positive for the Ki67, and 12% stained for cleaved caspase 3 (CC3), the apoptosis marker [87]. Co-expression of senescence and proliferation markers suggests that senescence is bypassed [88].

As described above, senescence is usually mediated by p53/p21 or p16/Rb [89, 90]. The presence of genetic mutations of oncogenes or tumor suppressors can cause cells to escape senescence and accelerate tumor progression. During PDA initiation and progression, a series of genetic changes such as *KRAS*, *P16*, *P53*, and *SMAD4* mutations release cancer cells from the limitations of senescence [67] (Fig. 2).

#### KRAS

*KRAS* is the first and most commonly detected genetic mutation in PDA [91, 92]. Once the *KRAS* is mutated, the inherent GTPase activity of RAS is disrupted and prevents GAPs from facilitating the conversion of GTP (active) to GDP (inactive), conferring permanent activation of the *KRAS* and its downstream pathways, leading to cell proliferation, migration, transformation, and adhesion survival [93]. PDA initiated by oncogenic *KRAS* is negative for senescence markers, including p16, p15^INK4b^, Dec1 and DcR2, while premalignant lesions are positive for those markers, suggesting that senescence exists in premalignant tumors but not in PDA [48]. p15^INK4b^ is a member of the INK4b family of cyclin-dependent kinase inhibitors. Both Dec1 and DcR2 are target genes of p53 in the induction of premature senescence [94]. Activation of the AKT signaling pathway, a downstream target of *KRAS*, suppresses *KRAS*-induced senescence, thus inducing a more aggressive PDA [95].

#### P16

Abrogation of the Rb/p16 tumor-suppressive pathway exists in almost all PDA. The central role of p16 in PDA development is evidenced by 80–95% of sporadic cases showing a loss of p16 function [96]. *P16* mutation is occasionally found in low-grade PanIN, but is observed frequently in high-grade PanIN [97]. p16 inactivation is an intermediate or late event in PanIN and PDA, and is the molecular switch for senescence evasion and unleashed *Kras*-induced malignancy [98]. In vitro experiments have shown that the induction of premature senescence of primary pancreatic duct epithelial cells preferentially depends on p16 and the expression of endogenous Kras in the cells can inhibit premature senescence by increasing Twist, an important regulator of embryogenesis, to eliminate *p16* expression [99, 100]. Inactivation of p16 can prevent PI3K-induced senescence in ADM lesions [101]. Other epigenetic regulators *Bmi1* and *Ring1B* cooperate with *p16* and play a critical regulatory role in avoiding senescence and facilitating malignant transformation in the pancreas [102, 103]. The up-regulation of p16 and the loss of p21 lead to increased senescence in ADM, further indicating p16 as a key regulator of pancreatic senescence.

#### P53

PDA shows a high frequency of *P53* inactivating mutations (50–70%) [104]. Ductal epithelial cells are origin for PDA, while *P53* mutations are required for generation of invasive PDA [105]. Endogenous Kras expression in the context of p53 leads to the formation of preinvasive PanIN and extensively metastatic PDA, which realistically recapitulates all extant features of human disease [106]. Studies have found that lack of p53 allows cells to escape *Kras*-induced senescence [71]. *P21* is an important target downstream of *P53* to mediate cell senescence [29]. The *LKB1/STK11* gene encodes a serine/threonine protein kinase, and its mutation causes Peutz-Jeghers syndrome [107]. The expression of LKB1 protein is significantly down-regulated in PDA [108]. Restoring the level of LKB1 can trigger the apoptosis of PDA cells [109]. Genetic restoration of *p53* in a RAS-driven murine liver carcinomas induces cell cycle arrest to promote tumor regressions by triggering differentiation and the upregulation of inflammatory cytokines [110]. LKB1 is directly recruited to the promoter of *P21* in a *P53*-dependent manner to participate in regulation of the cell cycle and apoptosis [111]. *Lkb1* acts as a tumor suppressor gene by inducing *p21* in PDA. The loss of LKB1 helps cells get rid of p21-mediated growth arrest and promotes the development of Kras-induced PDA [112].

Interestingly, the expression of p21 is low in caerulein-induced ADM, while p21 is highly expressed in injured pancreatic acinar cells. p21-null seems to enhance the caerulein-induced ADM formation by increasing β-catenin expression and relocalization. Down-regulation of p21 in ADM increases damage-induced senescence, suggesting that during pancreatitis, up-regulation of p21 can protect acinar cells from excessive accumulation of DNA damage and subsequent senescence [113]. Complement factor B (CFB) as an upregulated secreted protein contributes to PDA progression by promoting cellular senescence. Attenuation of endogenous CFB induces SAβgal-positive cells, cyclinD1, and p21 accumulation, which arrest cell cycle in the G1 phase [114]. Trefoil factor 1 (TFF1) is secreted factor accelerating tumorigenesis by antagonize the OIS process, in part by the activity of the p21 and EGFR-mediated pathway. During early tumorigenesis, TFF1 may mark the initial breakthrough of the OIS barrier. It may serve as a biomarker for the transition between senescence state and precancerous lesions. TFF1 expression elevates 5-fold in PanIN compared with normal duct epithelium, up-regulated expression of TFF1 is often observed in pancreatic malignancies [115, 116]. Cell cycle analysis appears that sub-G1 cells and senescent population increase when TFF1 is Knocked down, while its presence may allow a portion of premalignant cells to senescence evasion, then acquire epigenetic and genetic changes to accelerate tumorigenesis [117].

#### Inflammation

In addition to gene mutations, pancreatitis also seems to block the senescence of PDA precursor lesions, which exhibit certain cancer features resulting from interaction between senescent cells and their highly inflammatory microenvironment [66]. Inflammation not only promotes the formation of PanIN, but also induces the progression of PDA. *KRAS* can induce PDA development, provided that there is a pancreatitis. Inflammation-induced reprogramming can allow epithelial cells to avoid cell senescence [118]. In adult mice, pancreatitis abrogates senescence in low-grade PanIN but can reappear after the inflammatory response has subsided [66]. Senescence markers are found in the low-grade PanIN present in samples surgically removed from PDA patients. No such biomarkers are observed in pancreatic tumor cells [66]. Indeed, tissue injury, as it occurs in pancreatitis, weakens the defense mechanism posed by senescence and leads to its bypass by exocrine cells, which can then readily form PanIN [119]. Anti-inflammatory treatments might reverse tissue damage by maintaining the senescence phenotype of PanIN lesions and block PanIN progression [66, 120].

Mechanistically, *KRAS*-caused formation of reactive oxygen species (ROS) gradually increases during PDA progression. Increases in ROS drive the oncogenic transformation and tumor progression via Kras-mROS-PKD1-NF-κB signaling, which fosters PDA cell proliferation [121–123]. In the early stages of PDA, classical NF-κB/RELA signaling controls OIS and inhibits ADM and progression of PanIN by regulating the CXCL1/ CXCR2 and exerting the tumor-suppressive function. Once OIS fails, RELA promotes PDA initiation [124].

Genetic inactivation of the pancreatitis-inducing *Nupr1* impairs PanIN formation by regulating *Kras*-induced senescence. *Nupr1* is a chromatin-remodeling protein and can be strongly induced by acute pancreatitis [125, 126]. In mice, the oncogenic form of *Kras* fails to promote PanIN in the absence of Nupr1 [126]. Inactivation of Nupr1 increases pancreatic exocrine cell senescence. *Nupr1* also represents in concert with the oncogenic *Kras* to facilitate PanIN formation, aiding the transition from pre-tumor senescent PanIN lesions to mature PDA through bypassing senescence [127, 128].

#### Telomeric dysfunction

After acute pancreatitis, telomeric dysfunction impairs regeneration of the exocrine pancreas, which involves *p53* and *p21* independent mechanisms. It may act as a causal factor affecting progression in pancreatitis [129]. Telomeric dysfunction is a likely culprit for the genetic aberrations in PanIN, while reduction in telomere is found in all histological grades of PanIN as compared with normal ductal epithelium. Telomere length abnormalities are by far the most common early genetic instability in PanIN. Intact telomere presumably serves as caretakers of the pancreatic ductal genome. Critically shortened telomere may set the stage for accumulating progressive chromosomal abnormalities that facilitate the development toward invasive cancer and cause early death of patients with certain cancer types [70, 130]. During senescence, sustained DNA damage response signaling as a result of telomere damage leads to the induction of p53. p53 promotes the activation and accumulation of p21 and p16, thereby keeping pRB hypophosphorylated in its active form, while pRB is uniquely equipped to block DNA replication [131].

Telomerase activity is upregulated in all kinds of human solid tumors, containing cancers of the pancreas, prostate, colon, stomach, and so on [132]. Hiyama et al. reported that 95% of 43 PDA specimens showed telomerase activity but was absent in benign and premalignant tumors [133]. Upregulation of telomerase activity occurs relatively in advanced stage of PDA [134].

### Senescence and pro-tumor microenvironment

The microenvironment is another vital factor that needs to be assessed as far as senescence escape mechanisms are concerned. The cancer microenvironment is largely orchestrated by inflammatory cells, which is an important participant in the neoplastic process, fostering cancer growth, promoting proliferation and migration, and protecting cancer from immune attack [135]. Tumor cells and their supporting microenvironment resemble “seed and soil”, which are prerequisites for tumor development. Apart from growth retardation, senescent cells develop altered secretory activities, producing and secreting various cytokines and molecules, which are collectively known as the SASP, to create a fertile “soil” that reinforce cell cycle arrest, alter the microenvironment, and trigger immune surveillance [83, 136].

#### Cancer-associated inflammatory cells

The SASP components are complex, including inflammatory and immunomodulatory cytokines, which promote tumorigenesis, most commonly Interleukin-6 (IL-6) and -8 (IL-8). Specifically, IL-6 regulates Stat3 activation and could induce Nrf2 pathway to promote PDA cell proliferation and invasion. IL-8 mediates MMP-2 activity and promotes PDA progression via cancer–stromal interaction and through enhancing metastasis [137]. In addition, loss of *p53* or gain of oncogenic RAS markedly amplifies the promalignant paracrine activities of the SASP, thus facilitating tumor progression [47]. Senescent cells also secrete common effector molecules matrix metalloproteinases (MMPs). In some instances, the MMP-2 and MMP-3 produced by senescent cells can promote the invasion of various epithelial cells [11]. The histone deacetylase–associated protein SIN3B is also involved in senescence induction. SIN3B plays a tumor-promoting role in a PDA mouse model, whereas SIN3B is up-regulated in ADM and PanIN. Nevertheless, upon *Kras* activation, SIN3B enhances the expression of IL-1α, drives the production of SASP, and promotes the formation of pro-inflammatory tumor microenvironment. Besides, elimination of SIN3B weaken *Kras*-induced senescence and PanIN development [138]. Non-dividing senescent PanIN cells express cyclooxygenase-2 (Cox2), one of the pro-inflammatory factors released in tumors, and its activity strengthens the growth of PanIN lesions. Periodic treatment with the Bcl2 family inhibitors can target and decrease Cox2-expressing senescent cells and dramatically reduce tumorigenesis, suggesting that senescent PanIN cells provide essential support to tumor growth, at least in part, by their expression of the proinflammatory Cox2 enzyme [87]. Senescent cells promote macrophage senescence through the secretion of SASP, then, macrophages may influence other immune cells to evade tumor cell surveillance and senescent cell clearance [79].

#### Cancer-associated fibroblasts

There exist normal fibroblasts in the pancreas to suppress their neighboring aberrant hyperplasia and maintain normal gland connective tissue architecture. Fibroblasts can be irreversibly activated when exposed in the cancer lesion, termed as cancer-associated fibroblasts (CAFs), which provide fertile soil for tumor progression. CAFs are the main cellular component in PDA [139]. CAFs can produce a wide range of factors, e.g., TGF-β, Interferon (IFN), Interleukin (IL), chemokines, and other cytokines. CAFs can be converted into senescent fibroblasts due to natural aging, genetic mutations, and so on. Senescent fibroblasts and CAFs function via SASP factors [140]. SASP are not unique to senescence and can also be secretd in CAFs, which recapitulate almost all the characteristics of senescent fibroblasts. These includes a lot of activated signaling pathways and factors [140]. Caveolin-1 (CAV1), is expressed in CAFs, is strongly associated with cellular senescence. CAFs with high expression of CAV1 control the secretion of IL-6 and IL-8 by NF-κB signaling. Knockdown of CAV1 negatively affects the motility and invasiveness of PDA cells. Moreover, attenuated CAV1 is correlated with decrease p53 and increase cell cycle related genes G2/M checkpoint or Myc target genes [137].

#### Pancreatic stellate cells

Pancreatic stellate cells (PSC) are the major producer of extracellular matrix (ECM) in PDA [141]. PSC exist in a quiescent state in the healthy pancreas, whereas PSC are activated to promote the extensive matrix reaction and irreversible pathophysiological transformations during PDA development and progression [142, 143]. Senescent PSC strongly facilitates susceptibility of PSC to immune cell cytotoxicity and contributes to the development of pancreatic disorders. Areas staining positive for senescence overlaps with markers of PSC activation and dense infiltrates of immune cells [144]. Moreover, in the processes of inflammation, PSC and cell senescence proceed in a coupled fashion and occur in the same microenvironment as in pancreas [144]. In contrast to quiescent PSC, senescent stellate cells highly express IL-6, MMP-9, CXCL1, CXCL2, and CXCL3 as SASP factors [145, 146]. Sequestosome-1 (Sqstm1) correlates with activation status of PSC, while a lower level of Sqstm1 controls pro-inflammatory PSC and the transformation of senescent phenotype of PSC through increasing the ROS level, which in turn promotes PDA development [147].

Stromal heterogeneity Tumors co-opt the wound-healing response to provide an opportunistic stroma for their survival [148]. In PDA, stroma includes heterogeneous cell populations, which could account for over 90% of the total tumor volume. The effects of stroma in PDA are as manifold as its components, which interact with the tumor cells and play a bimodal role: as a tumor promoter or a tumor suppressor [149]. Stroma encircling the tumor cells restricts tumor growth and differentiation, which is thought to be an innate host response against tumorigenesis at early stage [150]. However, as tumor stroma continues to alter during tumor progression, certain changes in stromal behavior may shape an immunosuppressive and tumor-permissive microenvironment to invade adjacent structures and metastasize [135]. Senescence can remodel tumor surrounding tissues through modulating the character of adjacent stroma cells and cancer cells [151].

### Reversibility and reprogram of senescence

Telomerase cannot reverse the senescence growth arrest and lose their senescent morphology, neither by conferring telomerase activity nor by altering telomere length [89]. The 'irreversibility' of senescence seems to be true only if pivotal pathways that sustain senescence remain intact, whereas reversal into cell cycle appears feasible when the correct pathways are disrupted [152]. Some of the senescent cells may suffer genetic, epigenetic, microenvironment changes that give rise to regaining the ability to proliferate. Suppression of p53 expression leads to the abrogation of established state of senescence and immortalization [89, 153]. However, microinjection of anti-p53 antibodies into senescent fibroblasts is sufficient to reinitiate DNA synthesis and cell division efficiently, thus reversing senescence [154]. Moreover, senescence arrest is reversible only in the absence of p16 expression. Once the p16/pRB pathway is engaged, neither p53 nor pRB inactivation is sufficient for DNA synthesis [89]. Oncogenic RAS delivers a strong mitogenic signal and partially overcome the growth-inhibitory effects of p53 and p21, but not p16. Senescence cells with high p16 can’t be stimulated to synthesize DNA (S-phase) by RAS [89]. In addition, senescence can be reversed by inactivation of DDR [155, 156]; interleukin-dependent inflammatory network contributes to OIS in vivo; and attenuation of inflammatory cytokine can also lead to senescent cells reversion into the cell cycle [50]. There are intriguing evidences that senescence can be reestablished, after administration of rapamycin [95] or cessation of caerulein treatment [66].

Senescence may represent one form of in-depth dormancy, whereby cancer cells evade the direct cytotoxic impact of therapy and maintain the self-renewal capacity [157]. Cellular dormancy is often defined as a non-proliferating state, but reversible, while tumor dormancy indicates an equilibrium between cell proliferation and death that drive tumor growth without detectable size change [158]. Of note, senescence may be a prominent mechanism driving dormancy, because the senescence markers have been detected in dormant cancer cells [159]. Increasing studies support that senescence and dormancy shares some molecular signals, and senescence can regulate more mature dormancy mechanisms [158]. Autophagy can regulate the functional activity of senescent cells and is important in cancer dormancy [160]. Tumor microenvironment can regulate dormancy and tumor recurrence, determining the final fate of dormant cancer cells. SASP can awaken surrounding cells in a dormant state and reactivate signal for dormant cells to resume proliferation [161, 162].

## Management of PDA by targeting senescence

The fate of cells hinges on the temporal and spatial expression of key molecules, for example, cell cycle regulator genes, oncogenes and tumor suppressors. These molecules are closely associated with senescence, and indispensable in malignant transformation [163]. Cells undergoing senescence are typically a cell-autonomous response to genomic stress, in particular persistent DNA damage triggered by telomere shortening or expression of activated oncogenes [164]. Senescence is a physiologically fundamental and pathologically relevant program, up to the specific situation. The role of senescence in PDA is complex and can be double-edged sword, which could either inhibit or promote the carcinogenic process, depending on the PDA stage and the type of cells involved [164].

In the initiation stage of pancreatic tumorigenesis, pancreatic cells are exposed to protumoral effect leading to premalignant lesions, and oncogenic stimuli trigger the senescence phenotype, induce cell cycle arrest and prevent premalignant lesions progression [86], whereas the persistent presence of senescent cells in tissues can trigger tumor-promoting effects. In moderately advanced PanIN lesions and the late stage of PDA, pancreatitis and oncogene activation or tumor suppressor gene inactivation cause cells escape senescence, which tips the balance in favor of tumor development [12].

Chemotherapy and radiotherapy resistances are a persistent challenge that has plagued the efficacy and prognosis of PDA treatment. Surgery is regarded as the only treatment. However, most patients are diagnosed at advanced stage, leaving little chance of tumor resection. Even after surgical treatment, patients will usually relapse [165]. Search for non-surgical effective treatment of PDA has become an urgent task. Although senescence escapes early in the course of tumor evolution, the induction or reactivation of this program could be a realistic option in the treatment of cancer [166].

To this end, compounds that induce senescence have been developed, including CDK4/6 inhibitors, which have shown promise in pre-clinical and clinical studies. Leveraging mouse models of *Kras*-mutant lung cancer, inducing cellular senescence by combined MEK and CDK4/6 inhibition provokes SASP-dependent and natural killer (NK) cell surveillance program and tumor cell death [167]. A phase I study of the CDK4/6 inhibitor in pediatric patients with malignant rhabdoid tumors, neuroblastoma, and other solid tumors, displays an acceptable safety profile, dose-dependent pharmacokinetic characteristics, and preliminary signs of tumor stabilization [168]. In breast cancer, phase III randomized study has shown that the trial combination of CDK4/6 inhibitor therapy results in longer progression-free survival and a relatively higher quality of life [169]. In the mouse PDA study, CDK4/6 inhibitor treatment reduces tumor volume accompanied by a decrease in pRb and Ki67 as compared to no treatment. Therapy-induced senescent cells promote vascular remodeling through producing pro-angiogenic SASP, leading to enhanced drug delivery, efficacy of cytotoxic gemcitabine chemotherapy, and immune checkpoint blockade [166, 170]. CDK inhibition overcomes gemcitabine resistance in PDA phase I trial [171]. Although only a few results have been obtained in PDA to date, CDK inhibition could be a promising strategy for new, advanced therapeutic options to treat PDA [172].

Induction of senescence helps to eliminate tumor cells, but can also cause certain chemotherapy side effects. The accumulation of senescent cells in normal tissues as well as in pathological sites is largely detrimental. Many chemotherapies exhibit accelerated senescence in normal tissues and an increased risk of developing secondary tumors [173, 174]. The potentially harmful properties of chronically persisting senescent cells make their quantitative removal a prominent therapeutic priority to avoid deleterious side effects. Malignant progression of PanIN shows enrichment for genes regulated by Stat3 and Myc and has lower levels of genes repressed by NF-κB, while senescence escape in PanIN cells trigger malignant PDA and correlates with stem cell phenotype and EMT [175]. Removal of senescent cells may be effective as preventive therapy for the blocks of PanIN formation and progression to PDA [87]. Two-Step Senescence-Focused Cancer Therapies: pro-senescence followed by senescent cell removal could prevent tumor recurrence and maintain an anti-tumor tissue microenvironment [42, 176] (Fig. 2).

## Conclusions and future directions

Senescence is a dynamic and multi-step process in response to stress or developmental signals. Senescence plays beneficial or deleterious roles, depending on the trigger as well as the environment [177–179]. The biology of senescent cell populations could provide clues to long-sought mechanisms explaining why most malignancies occur in the elderly. Substantial evidence suggests that cellular senescence is an important contributor to the etiology, progression, and consequences of various diseases, including PDA [136]. Senescence limits development of pancreatic preneoplastic lesions by preventing the proliferation of precancerous cells, which is an evolutionary cancer-protective mechanism designed to enhance organismal adaptation. Cancer cells in PDA are unable to prevent their proliferation due to the loss of senescence effects, while chronically detrimental ramifications of senescence in tumor cells and microenvironment facilitate cancer progression. Evidently, the deleterious effects of senescent cells on PDA appear to outweigh their beneficial effects.

The senescence status of tumors is highly heterogeneous in PDA, while precision senescence-targeted treatment is a future research direction. As our understanding of the basic biology of PDA and senescence advances, there are still many issues that remain to be addressed. The characteristics of different senescent cell types, as well as the mechanisms underlying their effects on tumor cell phenotype, are still not understood and in vivo analyses are currently lacking. Further mechanistic understanding of molecular and physiological properties of senescence and their complex association with cancer will be the key to targeting rationally and effectively the senescent cells in the treatment of PDA.
